# Gastrointestinal bleeding due to an erosion of the superior mesenteric artery: an exceptional fatal complication of pancreatic pseudocyst

**Published:** 2012-07-04

**Authors:** Mahdi Bouassida, Mechaal Benali, Hédi Charrada, Mossaab Ghannouchi, Fathi Chebbi, Mohamed Mongi Mighri, Mohamed Msaddak Azzouz, Hassen Touinsi, Sadok Sassi

**Affiliations:** 1 Department of Surgery, Mohamed Tahar Maamouri Hospital, 8000 Mrazga, Nabeul, Tunisia; 2 Department of Ranimation, Mohamed Tahar Maamouri Hospital, 8000 Mrazga, Nabeul, Tunisia; 3 Department of Gastrology, Mohamed Tahar Maamouri Hospital, 8000 Mrazga, Nabeul, Tunisia

**Keywords:** Gastrointestinal bleeding, superior mesenteric artery, pancreatic pseudocyst, acute pancreatitis, emergency

## Abstract

The erosion of a pancreatic pseudocyst into an adjacent artery is a rare and highly lethal complication of pancreatitis with reported death rates of 12% to 40%. The majority of patients had bleeding from the splenic artery, the gastroduodenal artery and the anterior pacreaticoduodenal artery. Exceptionally, some cases with bleeding from the superior mesenteric artery, or hepatic artery were reported. We report the case of a 50 year old patient having a cataclysmic upper gastrointestinal bleeding due to an erosion of the superior mesenteric artery by a pancreatic pseudocyst, and discuss contemporary methods in diagnosis and management of the condition

## Introduction

The erosion of a pancreatic pseudocyst into an adjacent artery is a rare and highly lethal complication of pancreatitis with reported death rates of 12% to 40% [1]. Despite improvements in the diagnosis and management of pancreatic pseudocysts, the incidence of intracystic hemorrhage ranges from 6% to 17%. In recent reports, investigators have described the successful management of pancreatic pseudoaneurysms with endovascular techniques and have advocated percutaneous angiographic embolization as the preferred treatment modality. An operation should be reserved for actively and hemodynamically unstable patients. We report the case of a 50 year old patient having a cataclysmic upper gastrointestinal bleeding due to an erosion of the superior mesenteric artery by a pancreatic pseudocyst, and discuss contemporary methods in diagnosis and management of the condition.

## Patient and case report

**Figure 1:**
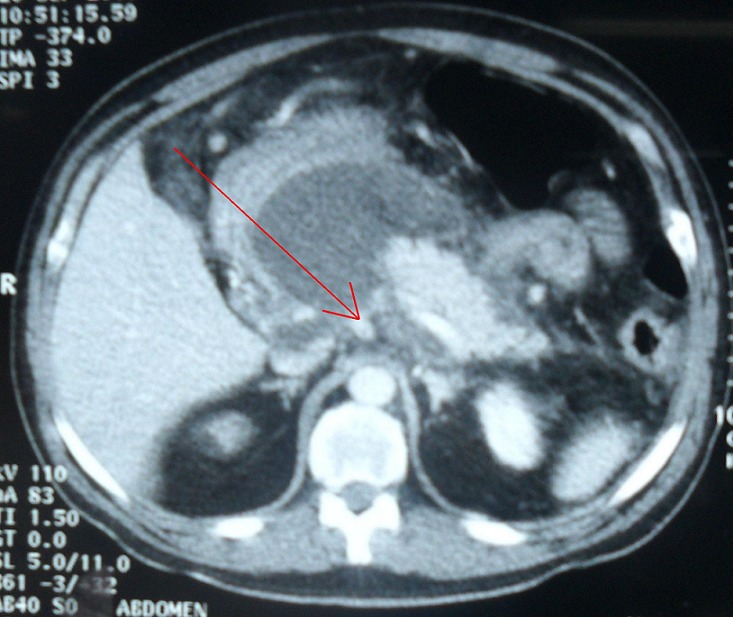
CT scan: A 135x58 mm well circumscribed unilocular cystic lesion, this lesion adhered to the posterior wall of the stomach and compressed the superior mesenteric artery (arrow)

A 50- year old man, with medical history of acute grade E pancreatitis, 3 months later, was admitted for epigastric pain and vomiting. Physical examination revealed a non-mobile mass in the upper abdomen. Laboratory serum results showed no abnormalities. The abdominal computed tomography (CT) scan revealed a 135x58 mm well circumscribed unilocular cystic lesion, this lesion adhered to the posterior wall of the stomach and compressed the superior mesenteric artery (Figure 1). We diagnosed a pancreatic pseudocyst. An endoscopic cystogastrostomy was planned but a cataclysmic hematemesis with shock occurred. Percutaneous angiographic embolization was not possible because of hemodynamic instability and an emergent laparotomy was carried out.

At laparotomy, by means of an anterior gastrotomy, a diagnosis of rupture of a bleeding pseudocyst into the posterior gastric wall was made. There was also an erosion of the superior mesenteric artery by the pseudocyst. We performed a suture of the bleeding point using a running 5-0 Prolène suture. The anterior gastrotomy was sutured as was the abdominal wall. Transfusion of 2000 mL of fresh blood was carried out, but the patient had a multiorgan failure and died one day after the intervention.

## Discussion

Pancreatic pseudocysts are common conditions following acute pancreatitis [2]. Bleeding is a rare complication, involving less than 5% of patients although carrying a mortality rate greater than 40% [3].

Three pathogenetic mechanisms of bleeding of pancreatic pseudocysts have been suggested [4]. The majority of patients had bleeding from the splenic artery (47%), the gastroduodenal artery (17%) and the anterior pacreaticoduodenal artery (16%) [5]. Exceptionally, some cases with bleeding from the superior mesenteric artery (such us our case), or hepatic artery were reported.

Pseudocysts may cause major vessel erosion with or without pseudoaneurysm formation which eventually may result in severe bleeding into the gastrointestinal tract (such us our case), retroperitoneum and peritoneal cavity [4]. The development of any bleeding complications unquestionably demands some sort of radiological or surgical management. In the management of massive bleeding from a pseudocyst, early diagnosis is essential. Dynamic bolus CT and angiography are considered to be the most useful means of finding a bleeding pseudocyst. Both methods have high accuracy and complement each other's findings. Angiography, in particular, has three important functions: (1) in the preoperative diagnosis for precise localization, (2) in angiographic embolization, and (3) in the preoperative identification of any unusual arterial anatomy [6].

Several surgical options have been proposed to control bleeding. A distal pancreatectomy and splenectomy is the most traditional procedure (if there is an erosion or a pseudoanevrysm of the splenic artery). Bleeding lesions in the head of the pancreas can be treated by pancreaticoduodenectomy [7].

Hemorrhage from vessels around the head or body may also be handled by ligation or oversewing the vessels. Bresler et al. reported that intracystic suture ligation and external drainage resulted in a good outcome [8]. However, suture and/or ligation of the bleeding point might be inappropriate in the presence of inflammatory, friable, necrotic, or bacterially contaminated tissue [4].

## Conclusion

The management of hemorrhagic complications of pancreatic pseudocysts remains a challenging problem with high morbidity and death. Operation and percutaneous angiographic embolization have complementary roles, and the optimal approach is determined by patient presentation. Percutaneous angiographic embolization is recommended as the initial treatment for hemodynamically stable patients. An operation should be reserved for actively bleeding, hemodynamically unstable patients; for failed embolization. Operative drainage should be considered after successful percutaneous angiographic embolization to avoid the development of secondary complications, particularly for large pseudoaneurysms. Careful follow-up is necessary because these patients frequently have pancreatic insufficiency or a new pseudocyst.
